# Disciplinary gender balance, research productivity, and recognition of men and women in academia

**DOI:** 10.1371/journal.pone.0293080

**Published:** 2023-12-14

**Authors:** Creso Sá, Summer Cowley, Bushra Shahrin, Colleen Stevenson, Ahmet Su

**Affiliations:** Department of Leadership, Higher, and Adult Education, Ontario Institute for Studies in Education, University of Toronto, Toronto, Ontario, Canada; National Taiwan University, TAIWAN

## Abstract

Gender disparities in science have become a salient concern for policy makers and researchers. Previous studies have documented a gender gap in research productivity and recognition in the sciences, and different reasons for this gap have been proposed. In this study, we examine four academic fields with different proportions of men and women in their population. We address the following questions: What is the relationship between the gendered make-up of a field and the productivity and recognition of men and women scientists in that academic field? What is the relationship between the publication patterns of men and women in different academic fields and their productivity and recognition? We find that gendered patterns of productivity and recognition favour men in man-dominated subfields (Mathematical Physics and Software Engineering), while women were more productive and highly cited in one woman-dominated subfield (Nursing), though not in another (Psychology). Nursing, a woman-gendered field, provides an interesting counterpoint to the most usual findings regarding gender disparities in academia. Our findings highlight the need to disaggregate academic fields and to bring to the forefront other disciplines that remain under investigated in analyses of gender gaps to potentially elucidate conflicting findings in the literature.

## Introduction

Gender gaps in science have become a salient policy concern over the past four decades. The underrepresentation of women in academic careers, as well as continuing obstacles to and disparities in their progress towards the upper echelons of science have attracted increasing attention, particularly across North America and Europe [1–5]. Research and policy debate over this period has contributed to a de-naturalization of science as a historically markedly gendered activity, prompting government research agencies, scientific institutions, academic societies, universities, and other research organizations to take steps to promote change.

Among the multiple manifestations of this issue are gender gaps in research productivity and recognition. Researchers have long investigated disparities between men and women in science in terms of publication and citation rates. Previous studies have shown that overall, men publish more, publish in higher impact factor (IF) journals, are more cited [6–10], and are proportionally more included in reference lists than women [11]. In some studies of citation patterns in the sciences, papers with men as first or last authors have been found to be cited more often, a pattern that persists when men’s self-citations are excluded [11], and also when including self-citations [12]. Further studies of the practice of self-citation have not always found gendered differences [13, 14].

Different patterns emerge, however, from studies focusing on subsets of research activity or narrow samples of scientists. For example, studies removing the top 10 per cent most prolific scientists from their analyses found that this eliminates gender gaps in productivity [15, 16]. Others find that scientists’ choice of research topics can explain some discrepancies in citations, as in some disciplines where more popular topics tend to be disproportionally investigated by men, thus generating greater attention and recognition from peers [17, 18]. The literature also shows that disparities in productivity can be traced to shorter career length and higher drop-out rates from science among women [12, 19], the effects of motherhood on career progression and publication trajectories [10], receiving less research funding [20], and being under-represented in many fields [21, 22]. It has also been found that women now have a greater chance than men to be elected into the National Academy of Science (NAS) and the American Academy of Arts and Science (AAAS) [23]. Discrepancies in the literature may also be due to location, as some studies focus on particular countries.

This study approaches the gender gap by foregrounding research fields, which can constitute a variety of environments with differing gender balances. The proportion of men and women scientists varies greatly across disciplines and subfields [24, 25], and a variety of explanations have been explored for these patterns. Women in STEM often receive fewer resources, and in resource-intensive research environments this impacts their ability to publish [17]. One study shows that women in math produced fewer publications, were less represented in top-ranked journals, and were more likely to leave their careers in the first ten years than men [7]. However, other work has found that once rank was taken into consideration, there were no significant gender differences in publications [21], and that while women were underrepresented within the top 10 per cent of scientists, there were no significant gender differences in productivity in the remaining 90 per cent [15, 16]. Research in management also revealed that more popular topics were mostly researched by men [18]. Overall, the gender bias in scientific publishing appears to be more salient in fields in which women are underrepresented [12], while studies that address specific disciplines, subfields, or research topics reveal inconsistent patterns [26].

The current study investigates differences between subfields that have different gendered compositions of researchers. Nygaard et al. [26] found that comparing publication data by scientific field, institutional affiliation, academic position, and age would cause gender differences to differ from the aggregate level. While women are underrepresented in most scientific disciplines and subfields [25], Nursing is one of the disciplines that has the highest proportion of women, at over half of all researchers [27]; conversely, Physics and Computer Science are two of the disciplines that have the fewest women authors [25]. However, research has shown that women perform equally to men in terms of publications in which they were the primary author and in terms of citations received in physics, among other disciplines [27]. Psychology is more gender-balanced [25, 27], but there are fewer women in the top 10 per cent of authors who receive nearly 50 per cent of the citations [28]. Previous research indicates that the proportion of men is increasing in previously woman-dominated subfields, and that the gender gap is not reducing quickly in heavily man-dominated subfields, such as Physics and Computer Science [25].

Against this background, this paper addresses the following questions: *what is the relationship between the gendered make-up of a field and the productivity and recognition of men and women scientists in that academic field*? *What is the relationship between the publication patterns of men and women in different academic fields and their productivity and recognition*? We sought to clarify how the gendered composition of different fields might have an impact on the relationship between gender, researchers’ journal selectivity and collaboration patterns, and their productivity and recognition. We focus on choice of journal selectivity and research collaboration as two major drivers of publication activity and subsequent citations. Preferences on whether and how to collaborate are also consequential in science, as highly cited articles are more likely to derive from collaborative research [29, 30]. Choice of publication venue matters too, because more prestigious journals tend to receive more citations [31], although this relationship has been decreasing in strength due to the ease of accessing articles online [32]. In general, men tend to publish more frequently in journals with higher impact factors [14], and women publish in less prestigious and less frequently cited formats [9]. Though there are studies with varied results indicating that women collaborate less in some samples [14] and more in others [33], several studies have concluded that women and men do not follow the same collaboration patterns, showing that women collaborate less internationally than men, which leads to fewer citations [6, 34]. Thus, drawing from previous studies, we hypothesize that men and women publish at different rates in higher ranking journals and collaborate at different rates with co-authors in general and with international co-authors in particular. These differences, in turn, would explain the gender gap in publication and citations. Expressed more formally, our hypotheses are:

*H1*: *Men’s productivity advantage is explained by patterns of research collaboration*.
*H1a*. *Men are more likely to engage in research collaboration*.
*H1b*. *Men are more likely to lead in inter-gender collaboration*.
*H2*. *Men’s citation advantage is explained by choice of publication venue*.
*H2a*. *Men are more likely to publish in highly ranked journals*.

We take a step further and hypothesize that the *gender make-up* of the fields in which authors work has a relationship to their publication choices and subsequent publication and recognition patterns. Namely, the gender balance of subfields affects researchers’ behaviors that contribute to or minimize the gender gap. This builds upon research on the effects of gender homophily in academia, which predicates that individuals connect with each other based on their demographic and social similarities, leading to homogenous professional networks [35, 36]. Previous research suggests homophily plays a role in co-publication and citations [36–41]. Put formally:

*H3*. *Gendered patterns of productivity and citations are related to the gender composition of academic fields*.
*H3a*. *Men are more prolific in Software Engineering and Mathematical Physics*.
*H3b*. *Men are more highly cited in Software Engineering and Mathematical Physics*.
*H3c*. *Women are more prolific in Psychology and Nursing*.
*H3d*. *Women are more highly cited in Psychology and Nursing*.

Our study puts forward a potential explanation for the at-times conflicting findings in the literature regarding gendered patterns of productivity and recognition. We argue that the gender composition of research fields creates different contexts for gender homophily, which in turn influences researchers’ publication choice, their patterns of collaboration, and peer recognition. We investigate these patterns across four research fields, building on previous observations that point to field norms having a greater impact on behavior such as international collaboration than gender [29]. To test our hypotheses, we gathered bibliometric data from four subfields that fell into two groups in terms of gender balance.

## Methods

We analyze four academic fields with differing gender make-ups: Psychology and Nursing–both woman-dominated; and Mathematical Physics and Software Engineering–both of which are within the man-dominated subfields of Physics and Computer Science [25]. These fields were selected based on their gender make-up and size of the publication output for the years selected. We used Holman et al. (2018) to determine the gender make-up, and selected Nursing and Psychology as comparable women-dominated areas. Physics and Computer Science were shown to be heavily men-dominated (Holman et al., 2018), but their volume of publication were not comparable in size to Nursing and Psychology, so we selected the subfields Mathematical Physics and Software Engineering to compare research fields of a similar scale. We compiled a dataset of journal articles from these disciplines. Using methods outlined by Thelwall [42, 43] as a guide, we analyze bibliometric data from English-language articles published by authors in Canada, the United States, Australia, New Zealand, the United Kingdom (England, Scotland, Wales and Northern Ireland), Ireland and France from 2006 to 2020. The search included publications that had authors listed from these English and French speaking countries for which we were able to find gendered name lists from authoritative sources. Publications that included at least one author from at least one of the countries listed were included. We selected 2006 as the start date for our period of interest to align with the first year that Web of Science started storing author’s full first names, rather than their initials only [44].

A bibliometric report of the articles (including source title, full names of all authors, address and email address, and number of times cited) was downloaded from Web of Science and was used as the main source of data for this study (see S1 Appendix). In addition to the data from the reports, we added columns to track author gender and number of co-authors. Author gender was estimated through cross-referencing the first names as listed in the articles (when present) to the name lists and censuses from each country. After the exclusion of articles for which no first name was listed for the first author, or for which gender of the first author could not be determined, the total number of articles was 206,421 (Table 1).

**Table 1:** Description of the sample.

Field	Total N (articles)	N gender ID’d	N woman first authors	% woman first authors	N unique first authors	N unique gender ID’d	% unique woman first authors
**Software Engineering**	55,119	18,856	8,239	21.2%	37,794	25,555	22%
**Psychology**	58,665	53,338	36,271	68%	32,807	29,123	69.6%
**Mathematical Physics**	61,546	39,540	6,545	16.6%	35,238	21,094	18.6%
**Nursing**	81,755	74,687	59,816	80.1%	47,635	42,751	79.8%

While there is no universally agreed upon process by which to determine author order in the sciences [45, 46], the first author cited is often the lead or corresponding author of the paper and also the one who did the majority of the work on the paper [47]. Vitale et al. [48] indicate that in the two highest ranking Nursing journals in Web of Science, the first author is typically the researcher who led the study. However, the determination of the amount of work done by each author varies and can be contested within research teams [45, 49, 50]. It should be noted that, despite variations in the meaning of authorship order, assessments of individual academics’ research productivity tend to focus on sole and first-authored publications [49].

All authors’ gender was determined via probabilistic estimations using the data sources described below for each country, a process common in the literature (e.g., [7, 24]) due to the impossibility of determining gender based on name alone. Articles where authors were identified only by initials were excluded. If the author’s first name was marked as one gender in more than 50 per cent of the instances in the databases, then they were identified as being of that gender. We selected the 50 per cent threshold in order to retain the greatest number of records in the dataset and avoid having an ambiguous gender category that would eliminate a number of the records we were analyzing. Additionally, we checked a random sample of 500 authors from each field (a total of 2000 authors) to confirm the reliability of the gender assignments by manually verifying their genders. The outcomes of this analysis indicated that the auto-assigned genders were correct at ~95% of the time, with little variability among the four fields (see Table 25 in S1 Appendix). As with other studies that determine gender based on first name, we encountered an issue related to non-Anglophonic names (Arabic, Middle Eastern) and non-Roman alphabet characters such as Chinese names. Based on these factors, we had to eliminate researchers whose names we could not match any gender with.

### US Census data

Lists of first names of respondents from the 1990 US Census were retrieved from the census administration website [51] as comma-delimited (.CSV) files. Each name in each list had been previously identified as either male or female, based on the self-identification of sex in the original census. A count of the number of respondents with each first name was also included in the Census data. We then combined the lists of female- and male-identified names into a single file, along with the counts of numbers of respondents with each first name. Each name was then given a weighted score for each gender based on the number of female and male respondents with that name.

### French name data

Lists of first names given to children born in France from 1900–2019 were downloaded as a comma-delimited (.CSV) file from the Institut national de la statistique et des études économiques of France [52]. Each name was identified as either male or female in this list. A count of the number of children given each name, each year, was included in the data.

### Australian name data

Lists of most popular baby names given to children born in Australia from 1944–2013, gathered by the Australian Attorney-General’s Department, were downloaded as comma-delimited (.CSV) files from data.gov.au [53]. Lists were separated by year and gender and were combined using an Ablebits Excel add-on.

### United Kingdom name data

Lists of baby names in England and Wales were gathered from the National Archives records of the Office for National Statistics [54–56]. Lists of the top baby names, categorized as those given to more than two babies, by gender and year, were downloaded in Excel (.XLS) files from The National Archives web archive. Lists were separated by year and gender and were combined using an Ablebits Excel add-on.

### Rate of inter-gender collaboration

To determine the rate of collaboration between women and men authors, we identified the gender of each of the author by the methods described above. Each author was assigned a dummy variable (0 or 1) depending on whether their name is associated with men (0) or women (1). Then at article level, we determined whether intergender collaboration took place for each article. If all authors in an article had the same gender (0 or 1), then there was no intergender collaboration in that article. If at least one author had a different gender, then there was collaboration in that article. Next, articles that had single authors were taken as no collaboration. Finally, at first author level, we determined for each first author how many times they authored an article that included intergender collaboration (Table 2). This resulted in the count data used for analysis.

**Table 2:** Variables and their definitions.

Variable Name	Definition
Total_collab auth1	The number of times the first author collaborated on a paper. The name of the first author of each article studied
intltotal_yes	Out of the articles the first author wrote, sum of the number of times they collaborated with someone internationally
intltotal_no	Out of the articles the first author wrote, sum of the number of times they did not collaborate with someone internationally
fem1	The gender of first author
gendcol_total_yes	Out of the articles the first author wrote, sum of the number of times they collaborated with someone of the opposite gender.
gendcol_total_no	Out of the articles the first author wrote, sum of the number of times they did not collaborate with someone of the opposite gender.
timescited_total	The sum of the number of citations the first author received in all their papers in the dataset.
if_high_count	The number of times the first author wrote in a high impact journal.
no_of_articles	The number of articles the first author wrote that are included in the dataset.
no_of_collaborators_any	The sum of the total number of collaborators of the first author in all their papers.

### Journal ranking

To determine the ranking of the journals in which the articles in our dataset were published, we first used the list of journal rankings by year at Scimago Journal Rank (SJR) to download rankings of all of the journals represented in our dataset from years 2006–2020. We chose the Scimago Journal Rank to rank journals by impact factor (IF), following Perera and Wijewickrema’s observations [57]. We then divided up the journal IFs into three intervals: 2006–2010, 2011–2015 and 2016–2020, and determined the average IF of each interval for each journal. Next, for each article, we used the average journal IF of the interval that the article’s publication date falls within. Finally, we divided up the articles into two categories based on their journal impact factors: high and not high. High/not high impact factor journals are defined as those with a Z-score above/below 1.65, corresponding to an alpha of 0.05.

### International and domestic collaboration

Using the Web of Science reporting tools, author location was determined by disaggregating the addresses reported for each author at time of publication. For those without an address listed, we used their email address to determine their affiliation and determined its location. We then added an additional variable for each coauthor (loc1, loc2, loc3, etc.) to store the name of country from which each author was working at the time of publication. We then calculated the rate of international to domestic collaboration (intcol) as a ratio of the number of authors from a different and from the same country as the lead author. If there was a single author in an article, it was determined that no collaboration took place. Finally, at first author level, it was determined how many times a first author appeared in an article that has international collaboration.

### Data analysis

We use a Poisson regression model with incidence rate ratios to measure the relationships between first-author gender, the number of times an article is cited, journal ranking, number of co-authors, international/domestic collaboration, cross-gender collaboration, number of articles published, and collaboration with co-authors. Data were analyzed using Stata 16 to run the Poisson regression models with interaction effects. Variables analyzed include number of times an article was cited, first author gender, number of co-authors, international/domestic/co-author collaboration, and whether an article was published in a high impact factor journal.

## Results

Overall, our analysis shows that there are gendered differences in publication patterns, productivity, and recognition in subfields with differing gender make-ups (Tables 3 and 4). There are nonetheless intriguing qualifications to this general finding. To start from the most expected findings, the man-dominated fields showed patterns consistent with most previous research that documents the gender gap in publications and citations. The baseline assumption that women were less likely to publish (0.93 times, p<0.001, as likely in Software Engineering, and 0.86 times, p<0.001, as likely in Mathematical Physics) and less likely to be cited compared to men was true (0.97 times, p<0.001, as likely in Software Engineering, and 0.70, p<0.001, times as likely in Mathematical Physics).

**Table 3:** Summary of results.

Hypotheses		SoftEng	Nursing	Math- Phys	Psych
H1: Men’s productivity advantage is explained by patterns of research collaboration	H1a. Men are more likely to engage in research collaboration	Yes	No	Yes	Yes
	H1b. Men are more likely to lead in inter-gender collaboration	No	Yes	No	Yes
H2. Men’s citation advantage is explained by choice of publication venue.	H2a. Men are more likely to publish in highly ranked journals.	Yes	---	---	Yes
H3. Gendered patterns of productivity and citations are related to the gender composition of academic fields.	H3a. Women are more prolific in Psychology and Nursing.	n/a	Yes	n/a	No
	H3b. Women are more highly cited in Psychology and Nursing.	n/a	Yes	n/a	No
	H3c. Men are more prolific in Software Engineering and Mathematical Physics.	Yes	n/a	Yes	n/a
	H3d. Men are more highly cited in Software Engineering and Mathematical Physics.	Yes	n/a	Yes	n/a

*”—” is used in the above table for hypotheses where results were insignificant and thus inconclusive.

**Table 4:** Summary of important coefficients in Poisson regressions.

Variables		Disciplines			
		Nursing	Psychology	SoftEng	Physics
The relationship between gender and …	# of publications	1.11**	0.90**	0.93**	0.87**
	# of citations	1.22**	0.79**	0.97**	0.76**
	# of publications in high IF journals	1.118	0.71**	0.7**	0.88
	Inter-gender collaboration	0.58**	0.75**	2.58**	3.59**
	Total collaboration/published with co-authors	1.18**	0.97	0.92**	0.96**
	Number of publications in high IF X intergender collaboration	0.70**	0.99	1.17	0.821
	Number of publications X gender collab.	0.72**	1.02	0.97	0.95**
	# of citations X inter-gender collaboration	0.72**	1.098*	0.97**	1.014**

=, ** p < .001; all other values = p<0.1 or not significant.

In what may explain those outcomes, their publication patterns differed markedly. In Software Engineering, women were 0. 70 times as likely to publish in journals with high IF compared to men (p<0.001). When women collaborated with men, their likelihood of publishing decreased (0.95 times as likely in Mathematical Physics, p<0.001). In Software Engineering, women had a lower likelihood of being cited if they collaborated with men (0.97 times as likely, p<0.001); however, as we hypothesized, women were less likely to publish with co-authors at all than men (0.92 times as likely, p<0.001).

There are a few qualifications to this overall pattern in the man-dominated fields. Notably, even considering their minority status within those fields and contrary to our expectation, women were more likely to lead inter-gender collaboration than men in both Software Engineering (2.58 times as likely, p<0.001) and Mathematical Physics (3.59 times as likely, p<0.001). Besides, there were no significant differences around publishing in high IF journals between genders in Mathematical Physics.

The sharpest contrast to the results from man-dominated fields came from Nursing, where women were more productive and more highly cited than men, in line with our hypotheses. In Nursing, women researchers are 1.11 times as likely to publish articles (p<0.001) and 1.22 times as likely to be cited (p<0.001). Women were 0.58 times as likely to collaborate across genders than men in Nursing (p<0.001). Moreover, women who published in higher IF journals were 0.90 times as likely to collaborate across genders (p<0.001), and such collaboration across genders was connected to a decreased likelihood of publishing in higher impact factor journals for women (0.7 times as likely, p<0.001).

Psychology provides an intriguing deviation from Nursing despite the relatively high representation of women. The general direction of findings seems to indicate that the field resembles the man-dominated disciplines in the expressions of the gender gap in publishing and citations. Overall, women are 0.94 times as likely to publish (p<0.001), and 0.79 times as likely to be cited as men in Psychology (p<0.001). In terms of the publication practices they followed, women were 0.75 times as likely to collaborate across genders as men (p<0.001), and collaboration with the opposite gender was associated with a decreased likelihood of publishing in higher impact factor journals for either men or women (0.89 times as likely), resembling the pattern seen in Nursing (p<0.001). Namely, men were more likely to make choices regarding their publication activity that are conducive to greater productivity and recognition, and indeed published more often and were more highly cited than women.

## Discussion

Our results show the overall suitability of our model, and the important role of research collaboration and choice of journal in explaining productivity and citations. Moreover, they highlight different patterns at play across the fields investigated with a higher and lower representation of women. Inequalities have been shown to be concentrated in specific research areas within disciplines; certain topics tend to be dominated by men, and these topics are frequently more highly researched and cited [17, 18]. Unlike with the man-dominated subfields we analyzed women were more productive and more highly cited than men in Nursing–but not in Psychology.

While some research shows that women publish less and gain less recognition than men in terms of citations and rank [6, 11, 27, 58] and that gender bias still exists against women [18, 22, 59], other work has shown that the gender gap is less present when certain factors are taken into account [16, 29]. Looking for an explanation of gendered discrepancies in scientific research, studies have shown that women publish less due to differences in career length and higher drop-out rates [19], receiving less funding [20], and access fewer academic positions [21, 22]. The gendered make-up of an academic field has an impact on the research behavior of women; however, a higher proportion of women in a field does not necessarily negate this bias, which is particularly illustrated in the contrast between Psychology and Nursing.

The differences we observe between Nursing and Psychology provide a contrast of disciplines with a higher representation of women, and reiterate that consideration of disciplinary norms, cultures, and dominant research practices remain central to analyses of the gender gap. Nursing, a woman-gendered field of research, teaching, and professional practice, provides an interesting counterpoint to the general findings of gender gaps in academia. These findings highlight the need to carefully disaggregate research fields in analyses of research productivity and recognition, rather than relying on broader catch-all categories (e.g. “Social Science”), in order to compare researchers operating within similar contexts and following the same publication norms. Moreover, these findings also suggest that we need a greater focus on fields and subfields with shifting gender compositions, in order to capture the potential effects of gender diversity on research activity and recognition, and to possibly explain the multiple possible relationships between them [60, 61]. The contrast between Nursing and Psychology in our results further suggest the need for future qualitative research to address the detailed and specific ways in which gender hierarchies are either perpetuated or overcome in fields where women are highly represented [62].

While gender inequities manifest at multiple levels, from individual to organizational to systemic [63], they may manifest differently across fields. For instance, research has suggested an association between field-specific beliefs about what it takes to be successful as a researcher and the representation of women [64]. Specifically, fields that culturally value ideas about “innate talent” as a condition for success were less likely to have a high proportion of women in their ranks. The mechanisms at play include discrimination based on gender stereotypes, which could arguably prevent field entry and also achievement through bias in resource allocation processes [59, 64]. In explaining these differences, future research might consider the internal dynamics of academic fields in comparative perspective, elucidating mechanisms of continuity and change in gender norms and research outcomes. As gender balances across academic fields shift and continue to change, further research must focus on both greater specificity in terms of research fields, as well as foreground other important social and cultural factors such as national and professional cultures, disciplinary norms, institutional structures, and academic beliefs that pervade disciplines.

## Supporting information

S1 Appendix (DOCX)
