# Supplementary material for: Disciplinary gender balance, research productivity, and recognition of men and women in academia
Source: PLoS One. 2023 Dec 14;18(12):e0293080. doi: 10.1371/journal.pone.0293080 (PMC10720991; doi:10.1371/journal.pone.0293080)
Supplement: S1 Appendix — (DOCX) [file pone.0293080.s001.docx]

# **Appendices**

**WoS column headings:**

Author full names - (full names of all the authors)

Source Title - (publication title)

Addresses - (addresses of all the authors)

Email Addresses - (email addresses of the authors)

Times Cited, All Databases - (number of times an article has been cited)

# **Poisson regression outputs**

## This section presents Poisson regression outputs of the four disciplines focused on in the study. Each section starts with the name of the discipline followed by the tables of the variables: total number of citations, number of published articles, total number of inter-gender collaboration, number of papers in high impact factor journals, and total number of first author’s collaboration.

## Software Engineering

**Table 5. Software Engineering Poisson regression results for total number of citations**

| timescited_total | IRR | | St.Err. | t-value | | p-value | [95% Conf Interval] | | | | Sig |
| --- | --- | --- | --- | --- | --- | --- | --- | --- | --- | --- | --- |
| First Author Female | 1 | | . | . | | . | . | | . | |  |
| Female | .97 | | .004 | -3.42 | | 0 | 0.98 | | 0.994 | | *** |
| total_collab | 1.142 | | .001 | 147.11 | | 0 | 1.14 | | 1.144 | | *** |
| fem1#co: base Male | 1 | | . | . | | . | . | | . | |  |
| Female | 0.963 | | .004 | -10.25 | | 0 | .0.956 | | .97 | | *** |
| no_of_articles | 1.068 | | .001 | 116.46 | | 0 | 1.067 | | 1.069 | | *** |
| fem1#co: base Male | 1 | | . | . | | . | . | | . | |  |
| Female | .0.987 | | .002 | --6.65 | | 0 | .983 | | .991 | | *** |
| no_of_collaborator~y | 1.017 | | 0 | 69.33 | | 0 | 1.016 | | 1.017 | | *** |
| fem1#co: base Male | 1 | | . | . | | . | . | | . | |  |
| Female | 1.018 | | .001 | 21.47 | | 0 | 1.017 | | 1.02 | | *** |
| gendcol_total_yes | 1.067 | | .001 | 52.22 | | 0 | 1.064 | | 1.07 | | *** |
| fem1#co: base Male | 1 | | . | . | | . | . | | . | |  |
| Female | .972 | | .003 | -9.40 | | 0 | .967 | | .978 | | *** |
| intltotal_yes | .929 | | .001 | -91.71 | | 0 | .927 | | .93 | | *** |
| fem1#co: base Male | 1 | | . | . | | . | . | | . | |  |
| Female | .972 | | .003 | -9.82 | | 0 | .967 | | .978 | | *** |
| IF_high_count | 1.206 | | .001 | 152.82 | | 0 | 1.203 | | 1.209 | | *** |
| fem1#co: base Male | 1 | | . | . | | . | . | | . | |  |
| Female | 1.119 | | .005 | 27.36 | | 0 | 1.11 | | 1.128 | | *** |
| Constant | 16.192 | | .028 | 1587.70 | | 0 | 16.137 | | 16.248 | | *** |
| Mean dependent var | | 25.005 | | | SD dependent var | | | 117.358 | |  |  |
| Pseudo r-squared | | 0.150 | | | Number of obs | | | 25292 | |  |  |
| Chi-square | | 266788.555 | | | Prob > chi2 | | | 0.000 | |  |  |
| Akaike crit. (AIC) | | 1509441.935 | | | Bayesian crit. (BIC) | | | 1509555.870 | |  |  |
| **** p<.01, ** p<.05, * p<.1* | | | | | | | | | | | |

**Table 6. Software Engineering Poisson regression results for number of published articles**

| no_of_articles | IRR | | St.Err. | t-value | | p-value | [95% Conf | | Interval] | | Sig |
| --- | --- | --- | --- | --- | --- | --- | --- | --- | --- | --- | --- |
| First Author Female | 1 | | . | . | | . | . | | . | |  |
| Female | .928 | | .014 | -4.82 | | 0 | .9 | | .957 | | *** |
| total_collab | 1.218 | | .004 | 63.49 | | 0 | 1.21 | | 1.225 | | *** |
| fem1#co: base Male | 1 | | . | . | | . | . | | . | |  |
| Female | .979 | | .011 | -1.90 | | .058 | .958 | | 1.001 | | * |
| no_of_collaborator~y | 1.007 | | .001 | 5.83 | | 0 | 1.005 | | 1.009 | | *** |
| fem1#co: base Male | 1 | | . | . | | . | . | | . | |  |
| Female | 1.002 | | .003 | 0.67 | | .503 | .996 | | 1.009 | |  |
| gendcol_total_yes | 1.059 | | .006 | 10.59 | | 0 | 1.048 | | 1.07 | | *** |
| fem1#co: base Male | 1 | | . | . | | . | . | | . | |  |
| Female | .973 | | .012 | -2.28 | | .022 | .951 | | .996 | | ** |
| intltotal_yes | .957 | | .003 | -12.89 | | 0 | .951 | | .963 | | *** |
| fem1#co: base Male | 1 | | . | . | | . | . | | . | |  |
| Female | 1.051 | | .012 | 4.32 | | 0 | 1.027 | | 1.075 | | *** |
| IF_high_count | 1.068 | | .008 | 8.82 | | 0 | 1.053 | | 1.084 | | *** |
| fem1#co: base Male | 1 | | . | . | | . | . | | . | |  |
| Female | 0.991 | | .023 | -0.40 | | .692 | .947 | | 1.037 | |  |
| Constant | 1.075 | | .008 | 10.20 | | 0 | 1.06 | | 1.09 | | *** |
| Mean dependent var | | 1.521 | | | SD dependent var | | | 1.506 | |  |  |
| Pseudo r-squared | | 0.134 | | | Number of obs | | | 25292 | |  |  |
| Chi-square | | 10063.344 | | | Prob > chi2 | | | 0.000 | |  |  |
| Akaike crit. (AIC) | | 64866.041 | | | Bayesian crit. (BIC) | | | 64963.700 | |  |  |
| **** p<.01, ** p<.05, * p<.1* | | | | | | | | | | | |

**Table 7. Software Engineering Poisson regression results for total number of inter-gender collaboration**

| gendcol_total_yes | IRR | | St.Err. | t-value | | p-value | [95% Conf | | Interval] | | Sig |
| --- | --- | --- | --- | --- | --- | --- | --- | --- | --- | --- | --- |
| First Author Female | 1 | | . | . | | . | . | | . | |  |
| Female | 2.582 | | .054 | 45.31 | | 0 | 2.478 | | 2.69 | | *** |
| total_collab | 1.269 | | .028 | 10.78 | | 0 | 1.215 | | 1.325 | | *** |
| fem1#co: base Male | 1 | | . | . | | . | . | | . | |  |
| Female | 1.168 | | .048 | 3.80 | | 0 | 1.078 | | 1.266 | | *** |
| no_of_collaborator~y | 1.04 | | .002 | 26.72 | | 0 | 1.037 | | 1.043 | | *** |
| fem1#co: base Male | 1 | | . | . | | . | . | | . | |  |
| Female | .981 | | .004 | -4.41 | | 0 | .973 | | .99 | | *** |
| no_of_articles | .886 | | .018 | -5.81 | | 0 | .851 | | .923 | | *** |
| fem1#co: base Male | 1 | | . | . | | . | . | | . | |  |
| Female | .908 | | .034 | -2.55 | | .011 | .843 | | .978 | | ** |
| intltotal_yes | .989 | | .007 | -1.71 | | .087 | .976 | | 1.002 | |  |
| fem1#co: base Male | 1 | | . | . | | . | . | | . | |  |
| Female | 1.015 | | .015 | 1.03 | | .302 | .986 | | 1.045 | |  |
| IF_high_count | .985 | | .016 | -0.99 | | .325 | .955 | | 1.016 | |  |
| fem1#co: base Male | 1 | | . | . | | . | . | | . | |  |
| Female | 1.089 | | .032 | 2.93 | | 0.003 | 1.029 | | 1.153 | | *** |
| Constant | .294 | | .004 | -88.90 | | 0 | .287 | | .302 | | *** |
| Mean dependent var | | 0.566 | | | SD dependent var | | | 0.854 | |  |  |
| Pseudo r-squared | | 0.152 | | | Number of obs | | | 25292 | |  |  |
| Chi-square | | 7793.253 | | | Prob > chi2 | | | 0.000 | |  |  |
| Akaike crit. (AIC) | | 43428.892 | | | Bayesian crit. (BIC) | | | 43526.551 | |  |  |
| **** p<.01, ** p<.05, * p<.1* | | | | | | | | | | | |

**Table 8. Software Engineering Poisson regression results for number of papers in high impact factor journals**

| IF_high_count | IRR | | St.Err. | t-value | | p-value | [95% Conf | | Interval] | | Sig |
| --- | --- | --- | --- | --- | --- | --- | --- | --- | --- | --- | --- |
| First Author Female | 1 | | . | . | | . | . | | . | |  |
| Female | .695 | | .049 | -5.12 | | 0 | .605 | | .799 | | *** |
| total_collab | 1.176 | | .023 | 8.15 | | 0 | 1.131 | | 1.223 | | *** |
| fem1#co: base Male | 1 | | . | . | | . | . | | . | |  |
| Female | 1.091 | | .18 | 0.53 | | .598 | .79 | | 1.507 | |  |
| no_of_collaborator~y | 1.028 | | .003 | 8.39 | | 0 | 1.021 | | 1.034 | | *** |
| fem1#co: base Male | 1 | | . | . | | . | . | | . | |  |
| Female | .985 | | .015 | -1.04 | | .297 | .957 | | 1.014 | |  |
| no_of_articles | 1.028 | | .017 | 1.69 | | .092 | .996 | | 1.061 | | *** |
| fem1#co: base Male | 1 | | . | . | | . | . | | . | |  |
| Female | .767 | | .106 | -1.91 | | .056 | .585 | | 1.007 | | *** |
| intltotal_yes | .972 | | .011 | -2.60 | | 0.009 | .951 | | .993 | | *** |
| fem1#co: base Male | 1 | | . | . | | . | . | | . | |  |
| Female | 1.238 | | .06 | 4.42 | | 0 | 1.126 | | 1.362 | | *** |
| gendcol_total_yes | 1.007 | | .02 | 0.34 | | .732 | .968 | | 1.047 | |  |
| fem1#co: base Male | 1 | | . | . | | . | . | | . | |  |
| Female | 1.171 | | .069 | 2.70 | | .007 | 1.044 | | 1.314 | | ** |
| Constant | .061 | | .002 | -98.83 | | 0 | .058 | | .065 | | *** |
| Mean dependent var | | 0.090 | | | SD dependent var | | | 0.421 | |  |  |
| Pseudo r-squared | | 0.074 | | | Number of obs | | | 25292 | |  |  |
| Chi-square | | 1235.741 | | | Prob > chi2 | | | 0.000 | |  |  |
| Akaike crit. (AIC) | | 15552.194 | | | Bayesian crit. (BIC) | | | 15649.853 | |  |  |
| **** p<.01, ** p<.05, * p<.1* | | | | | | | | | | | |

**Table 9. Software Engineering Poisson regression results for total number of first author’s collaboration**

| total_collab | IRR | | St.Err. | t-value | | p-value | [95% Conf | | Interval] | | Sig |
| --- | --- | --- | --- | --- | --- | --- | --- | --- | --- | --- | --- |
| First Author Female | 1 | | . | . | | . | . | | . | |  |
| Female | .921 | | .015 | -5.05 | | 0 | .892 | | .951 | | *** |
| no_of_articles | 1.082 | | .002 | 45.80 | | 0 | 1.079 | | 1.086 | | *** |
| fem1#co: base Male | 1 | | . | . | | . | . | | . | |  |
| Female | .979 | | .006 | -3.26 | | .001 | .966 | | .991 | | *** |
| no_of_collaborator~y | 1.029 | | .001 | 27.38 | | 0 | 1.026 | | 1.031 | | *** |
| fem1#co: base Male | 1 | | . | . | | . | . | | . | |  |
| Female | .994 | | .003 | -1.69 | | .092 | .987 | | 1.001 | | * |
| gendcol_total_yes | 1.079 | | .007 | 12.31 | | 0 | 1.066 | | 1.093 | | *** |
| fem1#co: base Male | 1 | | . | . | | . | . | | . | |  |
| Female | 1.056 | | .012 | 4.86 | | 0 | 1.033 | | 1.079 | | *** |
| intltotal_yes | 1.03 | | .004 | 8.29 | | 0 | 1.023 | | 1.038 | | *** |
| fem1#co: base Male | 1 | | . | . | | . | . | | . | |  |
| Female | 1.002 | | .012 | 0.13 | | .894 | .978 | | 1.026 | |  |
| IF_high_count | 1.067 | | .009 | 8.05 | | 0 | 1.051 | | 1.084 | | *** |
| fem1#co: base Male | 1 | | . | . | | . | . | | . | |  |
| Female | .981 | | .023 | -0.80 | | .424 | .937 | | 1.028 | |  |
| Constant | .954 | | .007 | -6.34 | | 0 | .941 | | .968 | | *** |
| Mean dependent var | | 1.341 | | | SD dependent var | | | 1.204 | |  |  |
| Pseudo r-squared | | 0.137 | | | Number of obs | | | 25292 | |  |  |
| Chi-square | | 9585.555 | | | Prob > chi2 | | | 0.000 | |  |  |
| Akaike crit. (AIC) | | 60535.969 | | | Bayesian crit. (BIC) | | | 60633.628 | |  |  |
| **** p<.01, ** p<.05, * p<.1* | | | | | | | | | | | |

## Psychology

**Table 10. Psychology Poisson regression results for total number of citations**

| timescited_total | IRR | | St.Err. | t-value | | p-value | [95% Conf Interval] | | | | Sig |
| --- | --- | --- | --- | --- | --- | --- | --- | --- | --- | --- | --- |
| First Author Female | 1 | | . | . | | . | . | | . | |  |
| Female | .787 | | .002 | -111.08 | | 0 | .784 | | .791 | | *** |
| total_collab | 1.12 | | .001 | 100.01 | | 0 | 1.118 | | 1.123 | | *** |
| fem1#co: base Male | 1 | | . | . | | . | . | | . | |  |
| Female | .765 | | .001 | -177.67 | | 0 | .763 | | .768 | | *** |
| no_of_articles | 1.08 | | .001 | 122.09 | | 0 | 1.078 | | 1.081 | | *** |
| fem1#co: base Male | 1 | | . | . | | . | . | | . | |  |
| Female | 1.093 | | .001 | 86.87 | | 0 | 1.091 | | 1.095 | | *** |
| no_of_collaborator~y | 1.014 | | 0 | 116.94 | | 0 | 1.014 | | 1.014 | | *** |
| fem1#co: base Male | 1 | | . | . | | . | . | | . | |  |
| Female | 1.028 | | 0 | 140.43 | | 0 | 1.028 | | 1.029 | | *** |
| gendcol_total_yes | .878 | | .001 | -126.56 | | 0 | .877 | | .88 | | *** |
| fem1#co: base Male | 1 | | . | . | | . | . | | . | |  |
| Female | 1.081 | | .001 | 57.04 | | 0 | 1.078 | | 1.084 | | *** |
| intltotal_yes | .931 | | .001 | -78.76 | | 0 | .929 | | .933 | | *** |
| fem1#co: base Male | 1 | | . | . | | . | . | | . | |  |
| Female | 1.112 | | .001 | 93.25 | | 0 | 1.109 | | 1.114 | | *** |
| IF_high_count | 1.088 | | .002 | 47.48 | | 0 | 1.085 | | 1.092 | | *** |
| fem1#co: base Male | 1 | | . | . | | . | . | | . | |  |
| Female | 1.318 | | .003 | 129.32 | | 0 | 1.312 | | 1.323 | | *** |
| Constant | 36.853 | | .064 | 2088.92 | | 0 | 36.728 | | 36.978 | | *** |
| Mean dependent var | | 45.728 | | | SD dependent var | | | 108.062 | |  |  |
| Pseudo r-squared | | 0.288 | | | Number of obs | | | 28413 | |  |  |
| Chi-square | | 839856.180 | | | Prob > chi2 | | | 0.000 | |  |  |
| Akaike crit. (AIC) | | 2081181.821 | | | Bayesian crit. (BIC) | | | 2081297.386 | |  |  |
| **** p<.01, ** p<.05, * p<.1* | | | | | | | | | | | |

## Table 11. Psychology Poisson regression results for number of published articles

| no_of_articles | IRR | | St.Err. | t-value | | p-value | [95% Conf | | Interval] | | Sig |
| --- | --- | --- | --- | --- | --- | --- | --- | --- | --- | --- | --- |
| First Author Female | 1 | | . | . | | . | . | | . | |  |
| Female | .943 | | .01 | -5.42 | | 0 | .923 | | .963 | | *** |
| total_collab | 1.211 | | .006 | 40.13 | | 0 | 1.2 | | 1.2223 | | *** |
| fem1#co: base Male | 1 | | . | . | | . | . | | . | |  |
| Female | .864 | | .005 | -26.21 | | 0 | .855 | | .873 | | *** |
| no_of_collaborator~y | 1.01 | | .001 | 12.91 | | 0 | 1.009 | | 1.012 | | *** |
| fem1#co: base Male | 1 | | . | . | | . | . | | . | |  |
| Female | 1.023 | | .001 | 18.19 | | 0 | 1.02 | | 1.025 | | *** |
| gendcol_total_yes | .907 | | .005 | -18.22 | | 0 | .897 | | .916 | | *** |
| fem1#co: base Male | 1 | | . | . | | . | . | | . | |  |
| Female | 1.018 | | .007 | 2.37 | | 0.018 | 1.003 | | 1.032 | | *** |
| intltotal_yes | .907 | | .004 | -11.33 | | 0 | .943 | | .959 | | *** |
| fem1#co: base Male | 1 | | . | . | | . | . | | . | |  |
| Female | 1.135 | | .007 | 21.44 | | 0 | 1.122 | | 1.148 | | *** |
| IF_high_count | .899 | | .01 | -9.40 | | 0 | .879 | | .919 | | *** |
| fem1#co: base Male | 1 | | . | . | | . | . | | . | |  |
| Female | 1.351 | | .019 | 21.76 | | 0 | 1.315 | | 1.388 | | *** |
| Constant | 1.433 | | .013 | 40.49 | | 0 | 1.408 | | 1.458 | | *** |
| Mean dependent var | | 1.828 | | | SD dependent var | | | 2.054 | |  |  |
| Pseudo r-squared | | 0.172 | | | Number of obs | | | 28413 | |  |  |
| Chi-square | | 17317.333 | | | Prob > chi2 | | | 0.000 | |  |  |
| Akaike crit. (AIC) | | 83098.376 | | | Bayesian crit. (BIC) | | | 83197.431 | |  |  |
| **** p<.01, ** p<.05, * p<.1* | | | | | | | | | | | |

**Table 12. Psychology Poisson regression results for total number of inter-gender collaboration**

| gendcol_total_yes | IRR | | St.Err. | t-value | | p-value | [95% Conf | | Interval] | | Sig |
| --- | --- | --- | --- | --- | --- | --- | --- | --- | --- | --- | --- |
| First Author Female | 1 | | . | . | | . | . | | . | |  |
| Female | .75 | | .01 | -21.30 | | 0 | .73 | | .77 | | *** |
| total_collab | 1.308 | | .025 | 14.14 | | 0 | 1.261 | | 1.358 | | *** |
| fem1#co: base Male | 1 | | . | . | | . | . | | . | |  |
| Female | .783 | | .019 | -10.31 | | 0 | .747 | | .82 | | *** |
| no_of_collaborator~y | 1.012 | | .001 | 16.60 | | 0 | 1.011 | | 1.014 | | *** |
| fem1#co: base Male | 1 | | . | . | | . | . | | . | |  |
| Female | 1.038 | | .001 | 32.80 | | 0 | 1.036 | | 1.04 | | *** |
| no_of_articles | .861 | | .015 | -8.59 | | 0 | .832 | | .891 | | *** |
| fem1#co: base Male | 1 | | . | . | | . | . | | . | |  |
| Female | 1.087 | | .024 | 3.71 | | 0 | 1.04 | | 1.136 | | *** |
| intltotal_yes | .954 | | .005 | -9.16 | | 0 | .945 | | .964 | | *** |
| fem1#co: base Male | 1 | | . | . | | . | . | | . | |  |
| Female | 1.167 | | .008 | 22.22 | | 0 | 1.152 | | 1.183 | | *** |
| IF_high_count | .838 | | .011 | -13.20 | | 0 | .817 | | .861 | | *** |
| fem1#co: base Male | 1 | | . | . | | . | . | | . | |  |
| Female | 1.333 | | .022 | 17.21 | | 0 | 1.29 | | 1.377 | | *** |
| Constant | .994 | | .011 | -0.60 | | .547 | .973 | | 1.015 | |  |
| Mean dependent var | | 1.123 | | | SD dependent var | | | 1.519 | |  |  |
| Pseudo r-squared | | 0.172 | | | Number of obs | | | 28413 | |  |  |
| Chi-square | | 14553.421 | | | Prob > chi2 | | | 0.000 | |  |  |
| Akaike crit. (AIC) | | 70180.964 | | | Bayesian crit. (BIC) | | | 70280.020 | |  |  |
| **** p<.01, ** p<.05, * p<.1* | | | | | | | | | | | |

**Table 13. Psychology Poisson regression results for number of papers in high impact factor journals**

| IF_high_count | IRR | | St.Err. | t-value | | p-value | [95% Conf | | Interval] | | Sig |
| --- | --- | --- | --- | --- | --- | --- | --- | --- | --- | --- | --- |
| First Author Female | 1 | | . | . | | . | . | | . | |  |
| Female | .706 | | .03 | -8.27 | | 0 | .65 | | .767 | | *** |
| total_collab | 1.13 | | .028 | 4.85 | | 0 | 1.075 | | 1.187 | | *** |
| fem1#co: base Male | 1 | | . | . | | . | . | | . | |  |
| Female | .751 | | .023 | -9.16 | | 0 | .706 | | .798 | | *** |
| no_of_collaborator~y | 1.02 | | .002 | 10.24 | | 0 | 1.016 | | 1.024 | | *** |
| fem1#co: base Male | 1 | | . | . | | . | . | | . | |  |
| Female | 1.045 | | .003 | 13.18 | | 0 | 1.038 | | 1.051 | | *** |
| no_of_articles | 1.056 | | .018 | 3.27 | | 0.001 | 1.022 | | 1.092 | | *** |
| fem1#co: base Male | 1 | | . | . | | . | . | | . | |  |
| Female | 1.127 | | .026 | 5.29 | | 0 | 1.078 | | 1.179 | | *** |
| intltotal_yes | .948 | | .015 | -3.48 | | 0.001 | .919 | | .977 | | *** |
| fem1#co: base Male | 1 | | . | . | | . | . | | . | |  |
| Female | 1.249 | | .023 | 12.03 | | 0 | 1.205 | | 1.295 | | *** |
| gendcol_total_yes | .885 | | .017 | -6.23 | | 0 | .852 | | .92 | | *** |
| fem1#co: base Male | 1 | | . | . | | . | . | | . | |  |
| Female | .99 | | .025 | -0.41 | | .68 | .941 | | 1.04 | |  |
| Constant | .097 | | .003 | -70.31 | | 0 | .091 | | .103 | | *** |
| Mean dependent var | | 0.116 | | | SD dependent var | | | 0.439 | |  |  |
| Pseudo r-squared | | 0.107 | | | Number of obs | | | 28413 | |  |  |
| Chi-square | | 2375.934 | | | Prob > chi2 | | | 0.000 | |  |  |
| Akaike crit. (AIC) | | 19907.194 | | | Bayesian crit. (BIC) | | | 20006.249 | |  |  |
| **** p<.01, ** p<.05, * p<.1* | | | | | | | | | | | |

**Table 14. Psychology Poisson regression results for total number of first author’s collaboration**

| total_collab | IRR | | St.Err. | t-value | | p-value | [95% Conf | | Interval] | | Sig |
| --- | --- | --- | --- | --- | --- | --- | --- | --- | --- | --- | --- |
| First Author Female | 1 | | . | . | | . | . | | . | |  |
| Female | 0.972 | | .011 | -2.53 | | 0.011 | .95 | | .993 | | *** |
| no_of_articles | 1.08 | | .003 | 25.36 | | 0 | 1.074 | | 1.086 | | *** |
| fem1#co: base Male | 1 | | . | . | | . | . | | . | |  |
| Female | .958 | | .004 | -10.57 | | 0 | .951 | | .966 | | *** |
| no_of_collaborator~y | 1.014 | | .001 | 20.70 | | 0 | 1.013 | | 1.016 | | *** |
| fem1#co: base Male | 1 | | . | . | | . | . | | . | |  |
| Female | 1.022 | | .001 | 18.42 | | 0 | 1.02 | | 1.024 | | *** |
| gendcol_total_yes | 1.009 | | .005 | 1.79 | | .074 | .999 | | 1.019 | |  |
| fem1#co: base Male | 1 | | . | . | | . | . | | . | |  |
| Female | .921 | | .006 | -11.75 | | 0 | .908 | | .934 | | *** |
| intltotal_yes | .985 | | .004 | -3.40 | | .001 | .976 | | .993 | | *** |
| fem1#co: base Male | 1 | | . | . | | . | . | | . | |  |
| Female | 1.106 | | .007 | 16.71 | | 0 | 1.093 | | 1.119 | | *** |
| IF_high_count | .872 | | .011 | -10.92 | | 0 | .851 | | .894 | | *** |
| fem1#co: base Male | 1 | | . | . | | . | . | | . | |  |
| Female | 1.357 | | .02 | 20.42 | | 0 | 1.318 | | 1.398 | | *** |
| Constant | 1.268 | | .012 | 25.22 | | 0 | 1.245 | | 1.292 | | *** |
| Mean dependent var | | 1.669 | | | SD dependent var | | | 1.888 | |  |  |
| Pseudo r-squared | | 0.175 | | | Number of obs | | | 28413 | |  |  |
| Chi-square | | 16938.819 | | | Prob > chi2 | | | 0.000 | |  |  |
| Akaike crit. (AIC) | | 79745.496 | | | Bayesian crit. (BIC) | | | 79844.551 | |  |  |
| **** p<.01, ** p<.05, * p<.1* | | | | | | | | | | | |

## Nursing

**Table 15. Nursing Poisson regression results for total number of citations**

| timescited_total | IRR | | St.Err. | t-value | | p-value | [95% Conf | | Interval] | | Sig |
| --- | --- | --- | --- | --- | --- | --- | --- | --- | --- | --- | --- |
| First Author Female | 1 | | . | . | | . | . | | . | |  |
| Female | 1.218 | | .004 | 58.57 | | 0 | 1.21 | | 1.226 | | *** |
| total_collab | .939 | | .001 | -50.99 | | 0 | .937 | | .941 | | *** |
| fem1#co: base Male | 1 | | . | . | | . | . | | . | |  |
| Female | 1.166 | | .002 | 90.00 | | 0 | 1.162 | | 1.17 | | *** |
| no_of_articles | 1.033 | | 0 | 98.02 | | 0 | 1.033 | | 1.034 | | *** |
| fem1#co: base Male | 1 | | . | . | | . | . | | . | |  |
| Female | .998 | | .001 | -1.90 | | .057 | .997 | | 1 | | *** |
| no_of_collaborator~y | .99 | | 0 | -30.48 | | 0 | .989 | | .99 | | *** |
| fem1#co: base Male | 1 | | . | . | | . | . | | . | |  |
| Female | 1.017 | | 0 | 42.87 | | 0 | 1.016 | | 1.018 | | *** |
| gendcol_total_yes | 1.253 | | .002 | 126.73 | | 0 | 1.249 | | 1.258 | | *** |
| fem1#co: base Male | 1 | | . | . | | . | . | | . | |  |
| Female | .717 | | .001 | -177.23 | | 0 | .715 | | .72 | | *** |
| intltotal_yes | 1.077 | | .001 | 75.50 | | 0 | 1.075 | | 1.079 | | *** |
| fem1#co: base Male | 1 | | . | . | | . | . | | . | |  |
| Female | .936 | | .001 | -48.96 | | 0 | .933 | | .938 | | *** |
| IF_high_count | 1.392 | | .005 | 100.69 | | 0 | 1.383 | | 1.401 | | *** |
| fem1#co: base Male | 1 | | . | . | | . | . | | . | |  |
| Female | .94 | | .003 | -17.42 | | 0 | .934 | | .947 | | *** |
| Constant | 11.686 | | .036 | 800.98 | | 0 | 11.616 | | 11.756 | | *** |
| Mean dependent var | | 17.835 | | | SD dependent var | | | 47.149 | |  |  |
| Pseudo r-squared | | 0.237 | | | Number of obs | | | 42352 | |  |  |
| Chi-square | | 426084.374 | | | Prob > chi2 | | | 0.000 | |  |  |
| Akaike crit. (AIC) | | 1373632.565 | | | Bayesian crit. (BIC) | | | 1373753.718 | |  |  |
| **** p<.01, ** p<.05, * p<.1* | | | | | | | | | | | |
|  | | | | | | | | | | | |

**Table 16. Nursing Poisson regression results for number of published articles**

| no_of_articles | IRR | | St.Err. | t-value | | p-value | [95% Conf | | Interval] | | Sig |
| --- | --- | --- | --- | --- | --- | --- | --- | --- | --- | --- | --- |
| First Author Female | 1 | | . | . | | . | . | | . | |  |
| Female | 1.106 | | .012 | 9.69 | | 0 | 1.084 | | 1.129 | | *** |
| total_collab | 1.024 | | .004 | 6.01 | | 0 | 1.016 | | 1.032 | | *** |
| fem1#co: base Male | 1 | | . | . | | . | . | | . | |  |
| Female | 1.153 | | .006 | 29.43 | | 0 | 1.142 | | 1.164 | | *** |
| no_of_collaborator~y | .976 | | .001 | -18.84 | | 0 | .974 | | .979 | | *** |
| fem1#co: base Male | 1 | | . | . | | . | . | | . | |  |
| Female | 1.025 | | .002 | 16.47 | | 0 | 1.022 | | 1.027 | | *** |
| gendcol_total_yes | 1.229 | | .007 | 34.05 | | 0 | 1.214 | | 1.243 | | *** |
| fem1#co: base Male | 1 | | . | . | | . | . | | . | |  |
| Female | .72 | | .005 | -51.36 | | 0 | .711 | | .729 | | *** |
| intltotal_yes | 1.046 | | .004 | 12.26 | | 0 | 1.038 | | 1.053 | | *** |
| fem1#co: base Male | 1 | | . | . | | . | . | | . | |  |
| Female | .912 | | .005 | -17.74 | | 0 | .903 | | .921 | | *** |
| IF_high_count | 1.237 | | .017 | 15.44 | | 0 | 1.204 | | 1.27 | | *** |
| fem1#co: base Male | 1 | | . | . | | . | . | | . | |  |
| Female | .873 | | .014 | -8.56 | | 0 | .846 | | .9 | | *** |
| Constant | 1.295 | | .012 | 27.42 | | 0 | 1.272 | | 1.319 | | *** |
| Mean dependent var | | 1.745 | | | SD dependent var | | | 2.196 | |  |  |
| Pseudo r-squared | | 0.151 | | | Number of obs | | | 42352 | |  |  |
| Chi-square | | 22318.646 | | | Prob > chi2 | | | 0.000 | |  |  |
| Akaike crit. (AIC) | | 125751.349 | | | Bayesian crit. (BIC) | | | 125855.194 | |  |  |
| **** p<.01, ** p<.05, * p<.1* | | | | | | | | | | | |

**Table 17. Nursing Poisson regression results for total number of inter-gender collaboration**

| gendcol_total_yes | IRR | | St.Err. | t-value | | p-value | [95% Conf | | Interval] | | Sig |
| --- | --- | --- | --- | --- | --- | --- | --- | --- | --- | --- | --- |
| First Author Female | 1 | | . | . | | . | . | | . | |  |
| Female | .575 | | .008 | -40.43 | | 0 | .56 | | .59 | | *** |
| total_collab | 1.099 | | .012 | 8.78 | | 0 | 1.076 | | 1.123 | | *** |
| fem1#co: base Male | 1 | | . | . | | . | . | | . | |  |
| Female | 1.244 | | .019 | 14.48 | | 0 | 1.207 | | 1.281 | | *** |
| no_of_collaborator~y | 1.017 | | .001 | 16.03 | | 0 | 1.015 | | 1.019 | | *** |
| fem1#co: base Male | 1 | | . | . | | . | . | | . | |  |
| Female | 1.029 | | .001 | 20.94 | | 0 | 1.026 | | 1.032 | | *** |
| no_of_articles | .959 | | .009 | -4.45 | | 0 | .941 | | .977 | | *** |
| fem1#co: base Male | 1 | | . | . | | . | . | | . | |  |
| Female | .761 | | .009 | -21.88 | | 0 | .743 | | .78 | | *** |
| intltotal_yes | 1.004 | | .004 | 1.08 | | 0.279 | .997 | | 1.012 | | *** |
| fem1#co: base Male | 1 | | . | . | | . | . | | . | |  |
| Female | .909 | | .006 | -15.18 | | 0 | .898 | | .92 | | *** |
| IF_high_count | 1.351 | | .024 | 17.11 | | 0 | 1.305 | | 1.398 | | *** |
| fem1#co: base Male | 1 | | . | . | | . | . | | . | |  |
| Female | .904 | | .019 | -4.74 | | 0 | .867 | | .943 | | *** |
| Constant | .889 | | .01 | -10.27 | | 0 | .869 | | .909 | | *** |
| Mean dependent var | | 0.710 | | | SD dependent var | | | 1.231 | |  |  |
| Pseudo r-squared | | 0.165 | | | Number of obs | | | 42352 | |  |  |
| Chi-square | | 16871.438 | | | Prob > chi2 | | | 0.000 | |  |  |
| Akaike crit. (AIC) | | 85626.732 | | | Bayesian crit. (BIC) | | | 85730.578 | |  |  |
| **** p<.01, ** p<.05, * p<.1* | | | | | | | | | | | |

**Table 18. Nursing Poisson regression results for number of papers in high impact factor journals**

| IF_high_count | IRR | | St.Err. | t-value | | p-value | [95% Conf | | Interval] | | Sig |
| --- | --- | --- | --- | --- | --- | --- | --- | --- | --- | --- | --- |
| First Author Female | 1 | | . | . | | . | . | | . | |  |
| Female | 1.118 | | .059 | 2.10 | | .036 | .1.007 | | 1.24 | | * |
| total_collab | .892 | | .02 | -5.20 | | 0 | .854 | | .931 | | *** |
| fem1#co: base Male | 1 | | . | . | | . | . | | . | |  |
| Female | 1.187 | | .039 | 5.16 | | 0 | 1.112 | | 1.267 | | *** |
| no_of_collaborator~y | .991 | | .005 | -1.63 | | .104 | .981 | | 1.002 | |  |
| fem1#co: base Male | 1 | | . | . | | . | . | | . | |  |
| Female | 1.032 | | .006 | 5.08 | | 0 | 1.02 | | 1.045 | | *** |
| no_of_articles | 1.023 | | .009 | 2.56 | | .01 | 1.005 | | 1.041 | | *** |
| fem1#co: base Male | 1 | | . | . | | . | . | | . | |  |
| Female | .984 | | .018 | -0.85 | | 0.395 | .949 | | 1.021 | | *** |
| intltotal_yes | 1.177 | | .016 | 12.00 | | 0 | 1.146 | | 1.208 | | *** |
| fem1#co: base Male | 1 | | . | . | | . | . | | . | |  |
| Female | .893 | | .016 | -6.19 | | 0 | .861 | | .925 | | *** |
| gendcol_total_yes | 1.318 | | .033 | 11.17 | | 0 | 1.256 | | 1.384 | | *** |
| fem1#co: base Male | 1 | | . | . | | . | . | | . | |  |
| Female | .697 | | .018 | -13.63 | | 0 | .662 | | .734 | | *** |
| Constant | .049 | | .002 | -63.31 | | 0 | .044 | | .053 | | *** |
| Mean dependent var | | 0.067 | | | SD dependent var | | | 0.313 | |  |  |
| Pseudo r-squared | | 0.063 | | | Number of obs | | | 42352 | |  |  |
| Chi-square | | 1366.765 | | | Prob > chi2 | | | 0.000 | |  |  |
| Akaike crit. (AIC) | | 20418.989 | | | Bayesian crit. (BIC) | | | 20522.834 | |  |  |
| **** p<.01, ** p<.05, * p<.1* | | | | | | | | | | | |

**Table 19. Nursing Poisson regression results for total number of first author’s collaboration**

| total_collab | IRR | | St.Err. | t-value | | p-value | [95% Conf | | Interval] | | Sig |
| --- | --- | --- | --- | --- | --- | --- | --- | --- | --- | --- | --- |
| First Author Female | 1 | | . | . | | . | . | | . | |  |
| Female | 1.182 | | .014 | 14.09 | | 0 | 1.155 | | 1.21 | | *** |
| no_of_articles | .995 | | .003 | -1.62 | | .105 | .989 | | 1.001 | |  |
| fem1#co: base Male | 1 | | . | . | | . | . | | . | |  |
| Female | 1.026 | | .004 | 6.69 | | 0 | 1.018 | | 1.033 | | *** |
| no_of_collaborator~y | .979 | | .001 | -16.47 | | 0 | .977 | | .982 | | *** |
| fem1#co: base Male | 1 | | . | . | | . | . | | . | |  |
| Female | 1.054 | | .002 | 36.22 | | 0 | 1.051 | | 1.057 | | *** |
| gendcol_total_yes | 1.269 | | .008 | 37.38 | | 0 | 1.253 | | 1.285 | | *** |
| fem1#co: base Male | 1 | | . | . | | . | . | | . | |  |
| Female | .746 | | .005 | -43.97 | | 0 | .736 | | .756 | | *** |
| intltotal_yes | 1.068 | | .004 | 17.53 | | 0 | 1.061 | | 1.076 | | *** |
| fem1#co: base Male | 1 | | . | . | | . | . | | . | |  |
| Female | .921 | | .006 | -13.60 | | 0 | .91 | | .932 | | *** |
| IF_high_count | 1.217 | | .019 | 12.69 | | 0 | 1.181 | | 1.255 | | *** |
| fem1#co: base Male | 1 | | . | . | | . | . | | . | |  |
| Female | .915 | | .017 | -4.90 | | 0 | .883 | | .948 | | *** |
| Constant | .969 | | .01 | -2.96 | | .003 | .948 | | .989 | | *** |
| Mean dependent var | | 1.362 | | | SD dependent var | | | 1.754 | |  |  |
| Pseudo r-squared | | 0.152 | | | Number of obs | | | 42352 | |  |  |
| Chi-square | | 20499.437 | | | Prob > chi2 | | | 0.000 | |  |  |
| Akaike crit. (AIC) | | 114263.772 | | | Bayesian crit. (BIC) | | | 114367.617 | |  |  |
| **** p<.01, ** p<.05, * p<.1* | | | | | | | | | | | |

## Physics

**Table 20. Physics Poisson regression results for total number of citations**

| timescited_total | IRR | | St.Err. | t-value | | p-value | [95% Conf Interval] | | | | Sig |
| --- | --- | --- | --- | --- | --- | --- | --- | --- | --- | --- | --- |
| First Author Female | 1 | | . | . | | . | . | | . | |  |
| Female | .756 | | .003 | -65.19 | | 0 | .749 | | .762 | | *** |
| total_collab | 1.021 | | .001 | 32.29 | | 0 | 1.02 | | 1.022 | | *** |
| fem1#co: base Male | 1 | | . | . | | . | . | | . | |  |
| Female | .811 | | .003 | -60.33 | | 0 | .805 | | .816 | | *** |
| no_of_articles | 1.046 | | 0 | 194.37 | | 0 | 1.045 | | 1.046 | | *** |
| fem1#co: base Male | 1 | | . | . | | . | . | | . | |  |
| Female | 1.151 | | .002 | 66.59 | | 0 | 1.147 | | 1.156 | | *** |
| no_of_collaborator~y | 1.031 | | 0 | 65.76 | | 0 | 1.03 | | 1.032 | | *** |
| fem1#co: base Male | 1 | | . | . | | . | . | | . | |  |
| Female | 1.035 | | .001 | 31.73 | | 0 | 1.033 | | 1.037 | | *** |
| gendcol_total_yes | .998 | | .001 | -2.02 | | 0.044 | .995 | | 1 | | *** |
| fem1#co: base Male | 1 | | . | . | | . | . | | . | |  |
| Female | 1.014 | | .003 | 5.74 | | 0 | 1.009 | | 1.019 | | *** |
| intltotal_yes | 1.011 | | .001 | 15.63 | | 0 | 1.009 | | 1.012 | |  |
| fem1#co: base Male | 1 | | . | . | | . | . | | . | |  |
| Female | .994 | | .002 | -3.31 | | 0.001 | .99 | | .998 | | *** |
| IF_high_count | 1.302 | | .003 | 112.31 | | 0 | 1.296 | | 1.308 | | *** |
| fem1#co: base Male | 1 | | . | . | | . | . | | . | |  |
| Female | 1.338 | | .008 | 4.24 | | 0 | 1.322 | | 1.354 | | *** |
| Constant | 21.68 | | .035 | 1917.68 | | 0 | 21.611 | | 21.748 | | *** |
| Mean dependent var | | 28.618 | | | SD dependent var | | | 85.501 | |  |  |
| Pseudo r-squared | | 0.186 | | | Number of obs | | | 21083 | |  |  |
| Chi-square | | 270565.987 | | | Prob > chi2 | | | 0.000 | |  |  |
| Akaike crit. (AIC) | | 1184906.971 | | | Bayesian crit. (BIC) | | | 1185018.358 | |  |  |
| **** p<.01, ** p<.05, * p<.1* | | | | | | | | | | | |

**Table 21. Physics Poisson regression results for number of published articles**

| no_of_articles | IRR | | St.Err. | t-value | | p-value | [95% Conf | | Interval] | | Sig |
| --- | --- | --- | --- | --- | --- | --- | --- | --- | --- | --- | --- |
| First Author Female | 1 | | . | . | | . | . | | . | |  |
| Female | .864 | | .014 | -9.29 | | 0 | .838 | | .891 | | *** |
| total_collab | 1.105 | | .003 | 43.14 | | 0 | 1.1 | | 1.11 | | *** |
| fem1#co: base Male | 1 | | . | . | | . | . | | . | |  |
| Female | .999 | | .011 | -0.06 | | .949 | .978 | | 1.021 | |  |
| no_of_collaborator~y | 1.003 | | .002 | 1.28 | | .199 | .999 | | 1.006 | |  |
| fem1#co: base Male | 1 | | . | . | | . | . | | . | |  |
| Female | 1.006 | | .005 | 1.21 | | .228 | .996 | | 1.015 | |  |
| gendcol_total_yes | 1.032 | | .004 | 7.20 | | 0 | 1.023 | | 1.04 | | *** |
| fem1#co: base Male | 1 | | . | . | | . | . | | . | |  |
| Female | .949 | | .009 | -5.77 | | 0 | .932 | | .966 | | *** |
| intltotal_yes | 1.04 | | .002 | 16.44 | | 0 | 1.035 | | 1.044 | | *** |
| fem1#co: base Male | 1 | | . | . | | . | . | | . | |  |
| Female | 1.032 | | .007 | 4.98 | | 0 | 1.019 | | 1.045 | | *** |
| IF_high_count | 1.006 | | .013 | 0.47 | | 0.636 | .98 | | 1.033 | | *** |
| fem1#co: base Male | 1 | | . | . | | . | . | | . | |  |
| Female | 1.107 | | .039 | 2.88 | | .004 | 1.033 | | 1.185 | |  |
| Constant | 1.462 | | .009 | 60.45 | | 0 | 1.444 | | 1.48 | | *** |
| Mean dependent var | | 1.874 | | | SD dependent var | | | 2.312 | |  |  |
| Pseudo r-squared | | 0.177 | | | Number of obs | | | 21083 | |  |  |
| Chi-square | | 13713.121 | | | Prob > chi2 | | | 0.000 | |  |  |
| Akaike crit. (AIC) | | 63653.986 | | | Bayesian crit. (BIC) | | | 63749.461 | |  |  |
| **** p<.01, ** p<.05, * p<.1* | | | | | | | | | | | |

**Table 22. Physics Poisson regression results for total number of inter-gender collaboration**

| gendcol_total_yes | IRR | | St.Err. | t-value | | p-value | [95% Conf | | Interval] | | Sig |
| --- | --- | --- | --- | --- | --- | --- | --- | --- | --- | --- | --- |
| First Author Female | 1 | | . | . | | . | . | | . | |  |
| Female | 3.588 | | .085 | 54.02 | | 0 | 3.426 | | 3.758 | | *** |
| total_collab | 1.023 | | .012 | 1.91 | | .056 | .999 | | 1.046 | | *** |
| fem1#co: base Male | 1 | | . | . | | . | . | | . | |  |
| Female | 1.052 | | .025 | 2.10 | | .035 | 1.003 | | 1.102 | |  |
| no_of_collaborator~y | 1.082 | | .003 | 28.87 | | 0 | 1.076 | | 1.088 | | *** |
| fem1#co: base Male | 1 | | . | . | | . | . | | . | |  |
| Female | .949 | | .005 | -10.65 | | 0 | .939 | | .958 | | *** |
| no_of_articles | .989 | | .01 | -1.11 | | .266 | .97 | | 1.008 | | *** |
| fem1#co: base Male | 1 | | . | . | | . | . | | . | |  |
| Female | 1.002 | | .022 | 0.08 | | .937 | .96 | | 1.045 | |  |
| intltotal_yes | .987 | | .006 | -2.96 | | .003 | .969 | | .994 | | * |
| fem1#co: base Male | 1 | | . | . | | . | . | | . | |  |
| Female | 1.079 | | .01 | 7.93 | | 0 | 1.059 | | 1.099 | | *** |
| IF_high_count | .816 | | .027 | -6.27 | | 0 | .765 | | .869 | | *** |
| fem1#co: base Male | 1 | | . | . | | . | . | | . | |  |
| Female | 1.34 | | .007 | 5.64 | | 0 | 1.21 | | 1.483 | | ** |
| Constant | .236 | | .004 | -92.42 | | 0 | .229 | | .243 | | *** |
| Mean dependent var | | 0.479 | | | SD dependent var | | | 0.984 | |  |  |
| Pseudo r-squared | | 0.191 | | | Number of obs | | | 21083 | |  |  |
| Chi-square | | 7848.946 | | | Prob > chi2 | | | 0.000 | |  |  |
| Akaike crit. (AIC) | | 33370.695 | | | Bayesian crit. (BIC) | | | 33466.169 | |  |  |
| **** p<.01, ** p<.05, * p<.1* | | | | | | | | | | | |

**Table 23. Physics Poisson regression results for number of papers in high impact factor journals**

| IF_high_count | IRR | | St.Err. | t-value | | p-value | [95% Conf | | Interval] | | Sig |
| --- | --- | --- | --- | --- | --- | --- | --- | --- | --- | --- | --- |
| First Author Female | 1 | | . | . | | . | . | | . | |  |
| Female | .882 | | .095 | -1.16 | | .247 | .714 | | 1.091 | | * |
| total_collab | .98 | | .018 | -1.09 | | 0.275 | .945 | | 1.016 | | *** |
| fem1#co: base Male | 1 | | . | . | | . | . | | . | |  |
| Female | 1.139 | | .122 | 1.22 | | .223 | .924 | | 1.405 | |  |
| no_of_collaborator~y | 1.111 | | .012 | 9.83 | | 0 | 1.088 | | 1.135 | | *** |
| fem1#co: base Male | 1 | | . | . | | . | . | | . | |  |
| Female | .893 | | .027 | -3.73 | | 0 | .841 | | .947 | |  |
| no_of_articles | 1.027 | | .013 | 2.15 | | 0.032 | 1.002 | | 1.053 | | *** |
| fem1#co: base Male | 1 | | . | . | | . | . | | . | |  |
| Female | 1.097 | | .08 | 1.28 | | .202 | .951 | | 1.265 | |  |
| intltotal_yes | .778 | | .024 | -8.12 | | 0 | .733 | | .827 | | *** |
| fem1#co: base Male | 1 | | . | . | | . | . | | . | |  |
| Female | 1.29 | | .078 | 4.21 | | 0 | 1.146 | | 1.452 | | *** |
| gendcol_total_yes | 1.057 | | .04 | 1.46 | | 0.144 | .981 | | 1.139 | | *** |
| fem1#co: base Male | 1 | | . | . | | . | . | | . | |  |
| Female | .821 | | .058 | -2.80 | | 0.005 | .715 | | .943 | | *** |
| Constant | .033 | | .001 | -77.23 | | 0 | .03 | | .036 | | *** |
| Mean dependent var | | 0.041 | | | SD dependent var | | | 0.249 | |  |  |
| Pseudo r-squared | | 0.039 | | | Number of obs | | | 21083 | |  |  |
| Chi-square | | 289.668 | | | Prob > chi2 | | | 0.000 | |  |  |
| Akaike crit. (AIC) | | 7240.587 | | | Bayesian crit. (BIC) | | | 7336.062 | |  |  |
| **** p<.01, ** p<.05, * p<.1* | | | | | | | | | | | |

**Table 24. Physics Poisson regression results for total number of first author’s collaboration**

| total_collab | IRR | | St.Err. | t-value | | p-value | [95% Conf | | Interval] | | Sig |
| --- | --- | --- | --- | --- | --- | --- | --- | --- | --- | --- | --- |
| First Author Female | 1 | | . | . | | . | . | | . | |  |
| Female | .956 | | .016 | -2.67 | | .008 | .925 | | .988 | | *** |
| no_of_articles | 1.04 | | .001 | 36.95 | | 0 | 1.038 | | 1.042 | | *** |
| fem1#co: base Male | 1 | | . | . | | . | . | | . | |  |
| Female | 1.004 | | .009 | 0.51 | | .611 | .987 | | 1.022 | |  |
| no_of_collaborator~y | 1.051 | | .001 | 41.16 | | 0 | 1.049 | | 1.054 | | *** |
| fem1#co: base Male | 1 | | . | . | | . | . | | . | |  |
| Female | .984 | | .004 | -4.49 | | 0 | .976 | | .991 | | *** |
| gendcol_total_yes | .985 | | .004 | -3.94 | | 0 | .978 | | .993 | | *** |
| fem1#co: base Male | 1 | | . | . | | . | . | | . | |  |
| Female | 1.027 | | .008 | 3.26 | | .001 | 1.011 | | 1.043 | | *** |
| intltotal_yes | 1.029 | | .003 | 10.19 | | 0 | 1.023 | | 1.035 | | *** |
| fem1#co: base Male | 1 | | . | . | | . | . | | . | |  |
| Female | 1.027 | | .007 | 4.14 | | 0 | 1.014 | | 1.04 | | *** |
| IF_high_count | .928 | | .013 | -5.44 | | 0 | .903 | | .953 | | ** |
| fem1#co: base Male | 1 | | . | . | | . | . | | . | |  |
| Female | 1.247 | | .046 | 5.98 | | 0 | 1.16 | | 1.341 | | *** |
| Constant | 1.095 | | .008 | 12.79 | | 0 | 1.08 | | 1.111 | | *** |
| Mean dependent var | | 1.516 | | | SD dependent var | | | 1.845 | |  |  |
| Pseudo r-squared | | 0.199 | | | Number of obs | | | 21083 | |  |  |
| Chi-square | | 13916.972 | | | Prob > chi2 | | | 0.000 | |  |  |
| Akaike crit. (AIC) | | 56011.050 | | | Bayesian crit. (BIC) | | | 56106.525 | |  |  |
| **** p<.01, ** p<.05, * p<.1* | | | | | | | | | | | |

**Table 25. Manual confirmation of auto-assigned genders through 500-author samples**

|  | Confirmed | Wrong | Not sure |
| --- | --- | --- | --- |
| Nursing | 476 (95.2%) | 14 (2.8%) | 10 (2%) |
| Physics | 472 (94.4%) | 8 (1.6%) | 20 (4%) |
| Psychology | 482 (96.4%) | 13 (2.6%) | 5 (1%) |
| Software Engineering | 473 (94.6%) | 13 (2.6%) | 14 (2.8%) |
| Total (N=2000) | 1903 (95.15%) | 48 (2.4%) | 49 (2.45%) |
